# Biodistribution of the saponin-based adjuvant Matrix-M™ following intramuscular injection in mice

**DOI:** 10.3389/fddev.2023.1279710

**Published:** 2023-11-06

**Authors:** Cecilia Carnrot, Berit Carow, Anna-Karin E. Palm, Eray Akpinar, Per-Henrik Helgesson, Ingrid Lekberg Osterman, Emelie Bringeland, Bryant Foreman, Nita Patel, Johan Bankefors, Louis Fries, Linda Stertman

**Affiliations:** ^1^ Adjuvant Immunology, Product Development, Novavax AB, Uppsala, Sweden; ^2^ Adjuvant Characterization, Product Development, Novavax AB, Uppsala, Sweden; ^3^ Product Design and Formulation, Product Development, Novavax AB, Uppsala, Sweden; ^4^ Vaccine Immunology, Novavax Inc., Gaithersburg, MD, United States; ^5^ Research and Development, Novavax Inc., Gaithersburg, MD, United States

**Keywords:** vaccine, NVX-CoV2373, draining lymph node, vaccine safety, COVID-19, mechanism of action

## Abstract

Novel adjuvants are extensively utilized in the development of safe and effective vaccines against emerging pathogens. Matrix-M™ adjuvant is a saponin-based adjuvant used in several active clinical development programs and in widespread use in the COVID-19 vaccine NVX-CoV2373. Here, we conducted a biodistribution study to better understand the mechanism of action and safety profile for Matrix-M™ adjuvant. Radiolabeled saponins or cholesterol were incorporated into Matrix-A™ particles, which represent 85% of Matrix-M™. Labeled Matrix-M™ adjuvant was given to mice by intramuscular injection with or without SARS-CoV-2 Spike protein. Radioactivity of the adjuvant components was quantified in local and systemic tissues at seven timepoints over a period of 1–168 h. The highest saponin levels were found at the 1-h timepoint at the injection site, in the draining (iliac) lymph nodes, and in urine. Saponins were rapidly cleared from these tissues, reaching very low levels by 48–72 h. Systemically, saponins were found at low levels in the plasma, kidneys, liver, and bone marrow, and were barely detectable in other investigated tissues. Cholesterol was also found at high levels at the injection site and in the draining lymph nodes. These levels declined rapidly at first, then plateaued at 24–48 h. Radiolabeled cholesterol was found at very low levels in other tissues at the earliest timepoints, until increasing and stabilizing after the 24-h timepoint, indicating entry into the endogenous cholesterol recycling pool. This study demonstrates a rapid distribution of Matrix-M™ adjuvant from the injection site to the draining lymph nodes, thus excluding a depot effect as central to the mechanism of action for this adjuvant. The diverging clearance patterns for saponins and cholesterol are suggestive of at least partial disassembly of the Matrix-particles, which has implications for the downstream effects of Matrix-M™ adjuvant on adaptive immune responses. Systemic exposure to toxicologically relevant tissues is very low.

## 1 Introduction

Vaccines are among the most successful and cost-effective ways to battle infectious diseases. Importantly, vaccines are also a crucial part of pandemic preparedness as novel pathogens emerge, such as the SARS-CoV-2 virus; the cause of the COVID-19 pandemic. Such vaccines must create a strong enough stimulus to drive the desired immune response and be clinically effective, while retaining acceptable tolerability and safety for widespread uptake. One promising approach is recombinant subunit vaccines, in which the use of highly purified, pathogen-specific recombinant proteins enables specific targeting of the immune response to relevant epitopes by robust and thermally stable formulations. However, such recombinant proteins are generally poorly immunogenic and need to be paired with an adjuvant to induce robust antibody responses. Vaccine adjuvants are compounds that can be used in vaccines to increase the magnitude and/or tailor the quality of the immune response to a particular antigen (Reed et al., 2013; Di Pasquale et al., 2015). Matrix-M™ adjuvant (Novavax AB, Uppsala, Sweden) is a novel adjuvant, which is a critical component of the NVX-CoV2373 vaccine that recently received authorization for use in humans by multiple regulatory agencies (among those the U.S. Food and Drug Administration and the European Medicines Agency) and is listed on the World Health Organization’s emergency use listing for COVID-19 vaccines (Keech et al., 2020; Heath et al., 2021; Dunkle et al., 2022). Matrix-M™ adjuvant is also included in several other candidate vaccines in the clinical developmental phase, including targeting seasonal influenza (alone and in combination with COVID-19), malaria, and Ebola Virus Disease (Fries et al., 2020; Datoo et al., 2021; Shinde et al., 2021; Shinde et al., 2022).

Matrix-M™ adjuvant is a saponin-based adjuvant, made with saponins from the Chilean soap bark tree (*Quillaja saponaria* Molina) that are formulated with cholesterol and phospholipids into cage-like nanoparticles (Lövgren and Morein, 1988). Two types of such nanoparticles, Matrix-A™ and Matrix-C™, each made up with a specific saponin fraction (Fraction-A; Fr-A, and Fraction-C; Fr-C), are mixed at a set ratio to form Matrix-M™ adjuvant (Lövgren Bengtsson et al., 2011). The main component is Matrix-A™, which comprises 85% of Matrix-M™.

Matrix-M™ adjuvant has antigen dose-sparing properties and promotes the induction of a balanced Th1/Th2 type CD4^+^ T cell immune response, driving the generation of IgG subclasses with different effector functions (Osterhaus and Rimmelzwaan, 1998; Rajput et al., 2007; Radosević et al., 2008; Madhun et al., 2009; Pedersen et al., 2011; Reimer et al., 2012; Magnusson et al., 2013; Reed et al., 2013; Di Pasquale et al., 2015). Importantly, vaccines adjuvanted with Matrix-M™ have been shown to broaden the antibody response to include cross-protective antibodies (Smith et al., 2017). In addition, Matrix-M™ adjuvantation of purified protein antigens can also induce antigen specific CD8^+^ T cell responses (Bengtsson et al., 2016; Tian et al., 2021; Rydyznski Moderbacher et al., 2022). Importantly, several large clinical trials have shown that vaccines adjuvanted with Matrix-M™ have an acceptable safety profile (Keech et al., 2020; Heath et al., 2021; Dunkle et al., 2022).

To date there is a limited understanding of the pharmacokinetics and biodistribution of saponin-based adjuvants in general and of Matrix-M™ adjuvant in particular. In addition to addressing these issues, results from biodistribution studies can help to further define the mechanism of action (MoA) and safety profile of Matrix-M™-adjuvanted vaccines. Thus, the objective of the present study was to investigate the biodistribution of Matrix-M™ adjuvant using radiolabeled saponin (Fr-A) or cholesterol incorporated into the Matrix-A™ particles, which make up 85% of the Matrix-M™ adjuvant. By a head-to-head comparison of Matrix-A™ particles made up with radiolabeled Fr-A or with radiolabeled cholesterol, we were able to discern the biodistribution patterns of these two critical components separately. In addition, we asked whether the presence of the rS nanoparticle antigen included in the NVX-CoV2373 vaccine affects the biodistribution and/or excretion of the saponins.

## 2 Materials and methods

### 2.1 Animals and materials

Female BALB/c mice (8–10 weeks old) and female/male CD-1 IGS mice (8 weeks old) were purchased from Charles River Laboratories (CRL, Germany). Adjuvanticity studies on the Matrix-M adjuvant modified for saponin radiolabeling were performed at the Swedish National Veterinary Institute (SVA; Uppsala, Sweden) and the biodistribution study was carried out at Charles River Discovery Research Services (Kuopio, Finland). All experiments were approved by regional independent animal experimentation ethical review boards (Uppsala djurförsöksetiska nämnd and National Animal Experiment Board of Finland, respectively). At termination, animals were either euthanized by cervical dislocation (SVA) or by deep anesthesia with pentobarbital (180 mg/kg, intraperitoneal, Charles River Discovery Research Services).

Matrix-M™ adjuvant is composed of two 40 nm large cage-like particles made from two separate saponin fractions, i.e., Matrix-A™ and Matrix-C™ (85% and 15%, respectively). The Matrix-A™ and -C™ particles are formed by formulating purified saponin fractions, Fr-A and Fr-C, respectively, from the tree *Q. saponaria* Molina with cholesterol and phospholipids (Lövgren and Morein, 1988).

**FIGURE 1:**
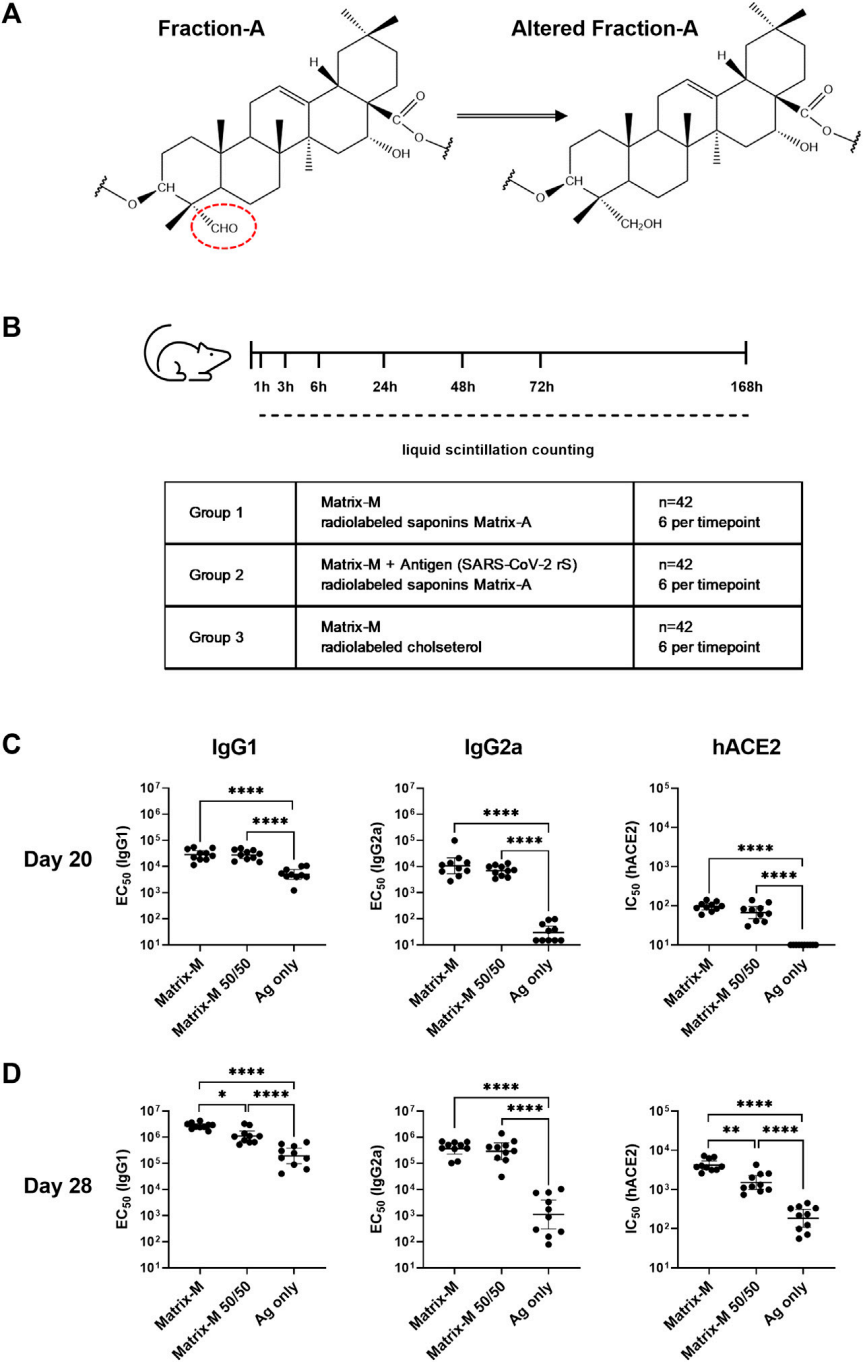
Adjuvanticity of Matrix-M™ adjuvant containing Matrix-A™ with partially modified saponins. **(A)** Representative structure displays Fraction-A and altered Fraction-A, in which the aldehyde in position C-23 on the triterpene core was reduced. **(B)** Study design for the biodistribution study describes the group composition and timepoints for measurements of radioactivity by liquid scintillation counting. **(C,D)** Mice were immunized subcutaneously with 1 μg rS with 5 μg Matrix-M, 1 μg rS with 5 µg Matrix-M (50/50), or 1 μg SARS-CoV-2 rS alone (“Ag only”) on Day 0 and Day 21. Serum samples obtained 20 days **(C)** and 28 days **(D)** after the primary immunization were evaluated for IgG1 and IgG2a antibody titers against rS protein and for hACE2 receptor-inhibiting antibody titers by ELISA. Individual titers are shown with each symbol, horizontal bars represent geometric mean titers and error bars represent 95% confidence intervals (CI). The data were analyzed by one-way ANOVA with Tukey’s multiple comparisons test. Statistically significant differences between groups are denoted with *, *p* < 0.05; **, *p* < 0.01; ****, *p* < 0.0001****, *p* < 0.0001. *n* = 10.

To track the biodistribution of saponins, all saponins in Fr-A were radiolabeled with tritium (^3^H). The aldehyde group on the quillaic acid triterpene aglycone (position C-23) was reduced to a hydroxyl group by tritium (^3^H)-labeled sodium borohydride [^3^H]-NaBH_4_ to obtain altered Fr-A material (Figure 1A). For synthesis of deuterium (^2^H)-labeled Fr-A utilized in the adjuvanticity evaluation of the modified material, sodium borodeuteride (NaBD_4_) was used as reducing agent. The altered Fr-A was mixed with unmodified Fr-A at 50:50 ratio and then formulated into Matrix-A™ adjuvant particles. This was subsequently mixed with unmodified Matrix-C™ at an 85:15 ratio to form Matrix-M™ adjuvant [Matrix-M (^3^H-Sap)]. To track the biodistribution of cholesterol, tritium labeled cholesterol [1-2-^3^H(N)] (Perkin Elmer) was used to formulate radiolabeled Matrix-A™ particles, which were used together with unmodified Matrix-C™ to form Matrix-M™ adjuvant [Matrix-M (^3^H-Chol)].

SARS-CoV-2 rS (construct BV2373, Novavax, Inc., Gaithersburg, MD) was constructed from the full-length, wild-type SARS-CoV-2 S glycoprotein based upon the GenBank gene sequence MN908947 (nucleotides 21563–25384). The native full-length S protein was modified by mutating RRAR to QQAQ (3Q) in the putative furin cleavage site located within the S1/S2 cleavage domain to become protease resistant. Two additional proline amino acid substitutions were inserted at positions K986P and V987P (2P) within the heptad repeat 1 (HR1) domain to stabilize SARS-CoV-2 rS in a prefusion conformation. The synthetic transgene has been engineered into the baculovirus vector (BV2373) for expression in Spodoptera frugiperda (Sf9) insect cells to produce SARS-CoV-2 rS proteins as previously described (Tian et al., 2021).

### 2.2 Immunization

#### 2.2.1 Adjuvanticity

BALB/c mice were immunized subcutaneously at the base of the tail, at a total volume of 100 µL/mouse, on days 0 and 21. Three animal groups (each *n* = 10) received 1 μg SARS-CoV-2 rS either unadjuvanted or in combination with 5 μg Matrix-M™ adjuvant or 5 μg Matrix-M™ adjuvant containing modified (^2^H-labeled) Matrix-A™ (Matrix-M™ 50/50). Blood was collected from the lateral tail vein of fully conscious animals on day 20 and on day 28. Serum was extracted from the blood and stored at −20 °C until analysis.

#### 2.2.2 Biodistribution

CD-1 IGS mice were immunized intramuscularly with a total volume of 40 µL into the right quadriceps femoris while anesthetized with isoflurane. Study groups and time points for termination are outlined in Figure 1B. Briefly, mice received a single dose of Matrix-M adjuvant (10 µg) containing radiolabeled saponins [Matrix-M (^3^H-Sap)], or Matrix-M adjuvant (10 µg) containing radiolabeled saponins and mixed with SARS-CoV-2 rS (1 µg) [Matrix-M (^3^H-Sap) + SARS-CoV-2 rS)], or Matrix-M adjuvant (10 µg) containing radiolabeled cholesterol and administered alone [Matrix-M (^3^H-Chol)] [each condition *n* = 42, 6 mice (3 females, 3 males) per time point]. The weight of the syringe was recorded before and after the dosing to measure the weight of the injected solution. The radioactivity of the dosing formulation (DPM/g) was determined by liquid scintillation counting (MicroBeta2, Perkin Elmer), and the injected (radioactive) dose (ID) was calculated and used to determine the normalized radioactivity as % of injected dose per gram tissue (as described below).

### 2.3 Blood and tissue sample collection for biodistribution

On the day of sacrifice, animals were weighed and then euthanized. Samples were then collected in tared tubes and weighed. First, blood samples were collected using cardiac puncture to prepare plasma for activity analysis. Immediately after blood sampling, the animals were perfused with heparinized saline (2.5 IU/mL) and tissue samples weighing a maximum of 200 mg were collected. The following samples were collected: injection site (quadriceps femoris muscle, QF), iliac lymph nodes (LN), inguinal LN, popliteal LN, plasma, urine, kidney, liver, intestines, spleen, lungs, bone marrow, heart, brain, axillary LN, mandibular LN, mesenteric LN, testes, ovaries, and uterus. Right and left LN were pooled.

### 2.4 Quantification of labeled saponins and cholesterol

For the scintillation analysis of total radioactivity, the tissue samples were mixed with 0.1 mL 0.9% saline and then homogenized (TissueLyser II, Qiagen) with 30 s-1 frequencies for 2 min at +4 °C. One mL Solvable (Perkin Elmer) was added to the homogenized tissues and samples were incubated for 2 h at + 60°C. Finally, the solubilized tissue homogenate was diluted at a ratio of 1:10 in UltimaGold (Perkin Elmer). Samples of lymph nodes were not homogenized but directly dissolved in 0.5 mL Solvable. The processed tissue samples were analyzed in a microplate counter (MicroBeta2; Perkin Elmer).

Radioactivity of the samples was measured as disintegrations per minute (DPM) and converted to % of injected radioactivity/g of tissue (normalized %ID/g; relative radioactivity/activity) by the following formula:

$$\left(\left(\frac{measured\;dose\;\left(DPM\right)}{injected\;solution\;\left(g\right)\times dose\;radioactivity\;\left(DPM/g\right)}\right)\times 100\right)/tissue\;weight\;\left(g\right)$$

The estimated limit of quantification (LOQ) was 703 DPM, which corresponds to a range of 0.0358–7.15 %ID/g tissue after normalization, depending on the mass of the tissue sample analyzed. Samples with DPM values < LOQ were set to 703 DPM before the %ID/g was calculated.

### 2.5 Anti-S IgG1 and IgG2a ELISA

Quantification of anti-S IgG1 and IgG2a antibodies in serum from day 20 and day 28 was performed by enzyme-linked immunosorbent assay (ELISA). Ninety-six-well MaxiSorp microplates (Nunc) were coated with 1.5 μg/mL SARS-CoV-2 rS protein (BV2373) in PBS overnight at 4°C. Individual sera were serially diluted in PBS containing 0.05% Tween-20 (PBS-T) and 1% bovine serum albumin (BSA) in deep-well plates. Serum samples were serially diluted 5-fold in 8 steps, starting at 1:300 or 1:1000 (day 20), 1:300 or 1:10,000 (day 28) for IgG1, and 1:15, 1:100 or 1:500 (day 20), 1:15, 1:100 or 1:1000 (day 28) for IgG2a. The samples were then added to the antigen-coated microtiter plates in singlicate (day 20) or duplicate (day 28) and incubated for 2 hours at room temperature. Pooled sera from untreated BALB/c mice (CRL, Germany) and sera from SARS-CoV-2 rS (with Matrix-M adjuvant) immunized mice were used as negative and positive controls, respectively. After washing with PBS-T, diluted HRP-linked secondary antibody, anti-IgG1 or -IgG2a (BIO-RAD Laboratories, Hercules, CA, United States), was added and incubated for 2 hours at room temperature. After washing, TMB substrate was added and incubated for 10 min, and then the reaction was stopped using 1.8 M sulfuric acid. The absorbance was measured at 450 nm (SpectraMax M3, Molecular Devices). The anti-S titers were calculated using a four-parameter logistic equation (SoftMax software v.6.5.1). The inflection point of the titration curve (EC50 value) was defined using a 4-parameter logistic equation, based on the curve fit estimated by SoftMax Pro. This was taken as the titer value. For a titer below the assay lower limit of detection (LOD), a titer of <15 (starting dilution) was reported and a value of “15” assigned to the sample to calculate the group mean titer.

### 2.6 hACE2 receptor inhibition ELISA

hACE2 receptor blocking antibody titers were determined by ELISA. Ninety-six-well plates were coated with 1.0 μg/mL SARS-CoV-2 rS protein overnight at 4°C. The coated wells were next blocked with StartingBlock™ (TBS) Blocking Buffer (ThermoFisher Scientific) for 1 h at room temperature. Mouse sera were serially diluted 2-fold starting with a 1:20 dilution and were added to coated wells and incubated for 1 h at room temperature. After washing, 30 ng/mL of histidine-tagged hACE2 (Sino Biologics, Beijing, CN) was added to wells and incubated for 1 h at room temperature. HRP-conjugated anti-histidine IgG was added and incubated for 1 h, followed by addition of TMB substrate. The absorbance was measured at 450 nm with a SpectraMax Plus plate reader (Molecular Devices, Sunnyvale, CA, United States) and data were analyzed with SoftMax Pro 6.5.1 GxP software. Serum antibody titer at 50% inhibition (IC50) of hACE2 to SARS-CoV-2 rS protein was then determined in the SoftMax Pro program. For a titer below the assay lower limit of detection (LOD), a titer of half the value of the starting dilution (value of “10”) was assigned to the sample to calculate the group mean titer.

### 2.7 Statistical analysis

Data were analyzed statistically using GraphPad Prism software (version 9). For IgG1, IgG2a and hACE2 data, the geometric mean titer (GMT) with associated 95% confidence intervals (CI) were calculated by group, and the mean values of the log10-transformed titer measurements were compared between groups using a one-way ANOVA with Tukey’s multiple comparisons test. Comparisons of %ID/g or %ID/mL between the groups at different timepoints were analyzed by two-way ANOVA followed by Tukey’s test. A *p*-value of <0.05 was considered statistically significant.

## 3 Results

### 3.1 Adjuvanticity of Matrix-M™ containing Matrix-A™ with partially modified saponins

Tracking the biodistribution of saponins after immunization with Matrix-M™ adjuvant was enabled by radiolabeling Fr-A by reduction of the aldehyde substituent on the quillaic acid triterpene aglycone (position C-23) (Figure 1A). The adjuvanticity of unmodified Matrix-M™ adjuvant in comparison to Matrix-M™ formulated from 50% altered and 50% unaltered Fr-A material (Matrix-M™ 50/50) was evaluated in a 2-dose immunization scheme with SARS-CoV-2 rS antigen in BALB/c mice to confirm retention of adjuvanticity (Figure 1B). At day 20 post primary immunization, similar titers of SARS-CoV-2 rS-specific IgG1 and IgG2a as well as similar titers of functional human angiotensin converting enzyme two (hACE2) receptor blocking antibodies were found in sera of both adjuvanted groups (Figure 1C); with both clearly superior to unadjuvanted antigen. At day 28, the IgG1 anti-rS, along with the hACE2 receptor inhibiting antibody response, were somewhat reduced in the group that had received SARS-CoV-2 rS adjuvanted with Matrix-M™ 50/50 (Figure 1D), while IgG2a anti-rS titers remained essentially the same in both adjuvanted groups. The comparison to unadjuvanted SARS-CoV-2 rS immunization showed an adjuvant effect of both unmodified Matrix-M™ and Matrix-M™ 50/50 regarding rS-specific IgG1 and IgG2a as well as hACE receptor blocking antibodies at day 20 and day 28. Thus, the clear, if marginally reduced, adjuvant function of Matrix-M™ 50/50 as assessed by humoral immunity suggests the suitability of modified Matrix-M™ 50/50 to represent Matrix-M™ adjuvant to follow the biodistribution of saponins. Of interest, the Matrix-M™ containing labeled saponins showed no reduction in its capacity to induce antigen-specific IgG2a responses, a marker of the known tendency of Matrix-M™ to support strongly Th1-biased T cell responses.

### 3.2 Local biodistribution

To study the local biodistribution and particle integrity of Matrix-M™ adjuvant after intramuscular injection in CD-1 mice, radioactivity in the quadriceps femoris muscle (QF; injection site) and lymph nodes (LN) draining the hind limb was analyzed at seven timepoints starting at 1 h and continuing to 168 h (7 days) after injection of Matrix-M™ adjuvant containing either radiolabeled saponins (+/-without SARS-CoV-2 rS antigen) or cholesterol (Figure 1B).

**FIGURE 2:**
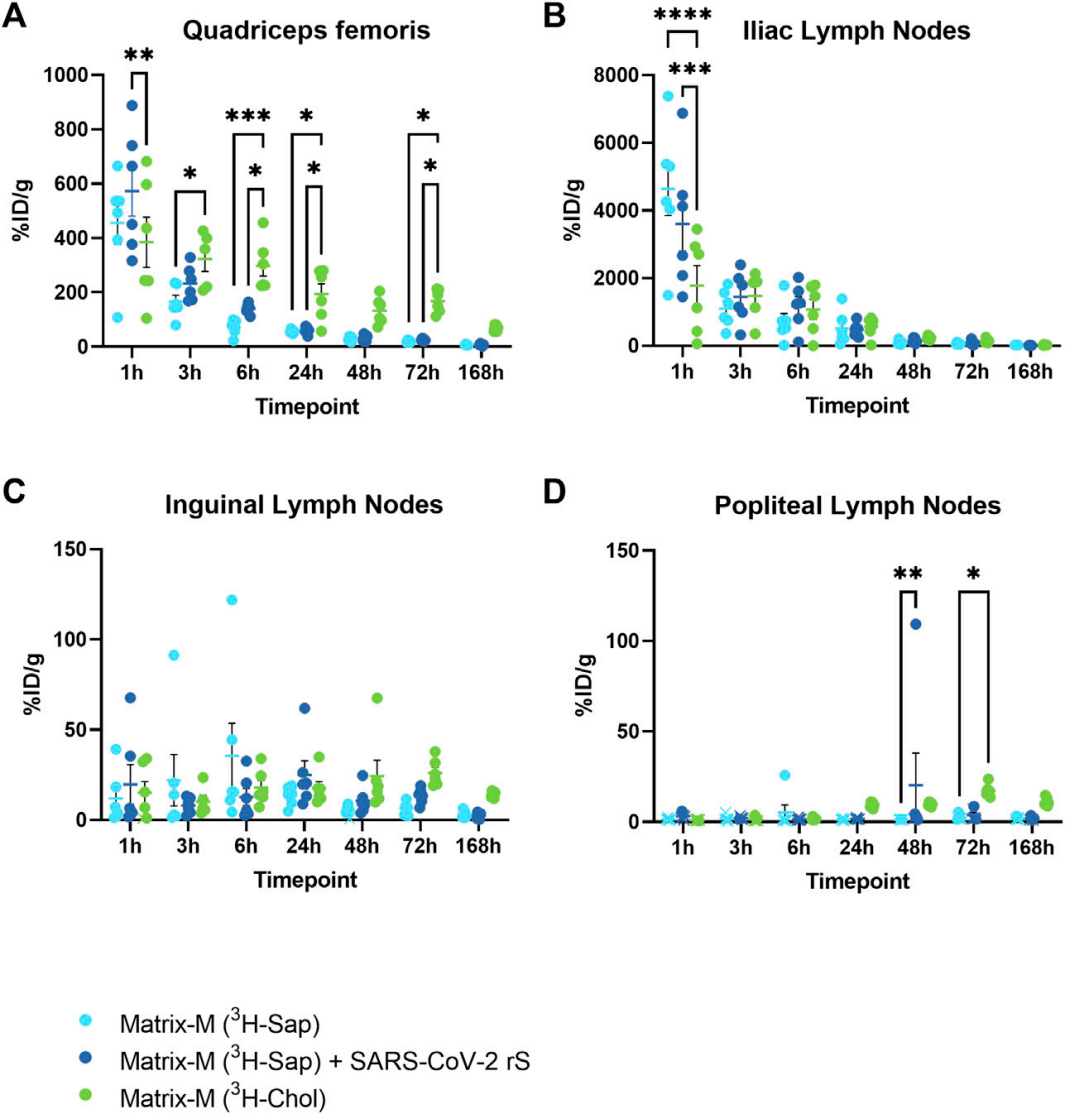
The distribution of saponins and cholesterol at the injection site and to the local draining lymph nodes. Matrix‐M™ adjuvant was formulated with either radiolabeled ^3^H-saponin or ^3^H-cholesterol, the former administered with or without SARS-CoV-2 rS antigen. The activity was measured in the Quadriceps femoris muscle **(A)** (injection site) and iliac **(B)**, inguinal **(C)**, and popliteal **(D)** lymph nodes (pooled left and right) by liquid scintillation and expressed as % of injected dose (ID) per gram tissue. Activity (DPM) below the LOQ (703 DPM) was set to 703 DPM and the %ID/g tissue was determined accordingly. Each datapoint represents an individual animal, the horizontal bar denotes the mean+/-standard deviation. Datapoints below the LOQ are shown as X. The data were analyzed separately for each tissue and timepoint by two-way ANOVA with Tukey’s multiple comparisons test. Statistically significant differences between groups are denoted with *, *p* < 0.05; **, *p* < 0.01; ***, *p* < 0.001; ****, *p* < 0.0001. *n* = 5–6.

One hour post injection (p.i.), a high percentage of injected radioactive dose per gram tissue (%ID/g) for both saponins and cholesterol was detected at the injection site and in the iliac LN (Figures 2A, B; Supplementary Figure S1A, B). Distribution to the iliac LN was substantial as compared to distribution to the inguinal and popliteal LN, thus indicating the iliac LN as the primary draining LN (dLN) after QF injection (Figures 2B, C). Following labeled saponins and cholesterol in the QF and the iliac LN over the time course, both showed a fast decline starting from 1 h p.i. At 48 h p.i., saponins reached low levels in both the QF and the iliac LN and remained low for the remainder of the experiment (Figures 2A, B; Supplementary Figure S1A). At the injection site, cholesterol counts showed a slower decline compared to saponin counts, leading to significantly higher cholesterol than saponin levels starting at 3–6 h p.i., suggesting that Matrix particles had been at least partially disassembled at these timepoints (Figure 2A). Presence or absence of SARS-CoV-2 rS antigen was found to have no effect on the local biodistribution of saponins (Figure 2).

### 3.3 Systemic biodistribution

**FIGURE 3:**
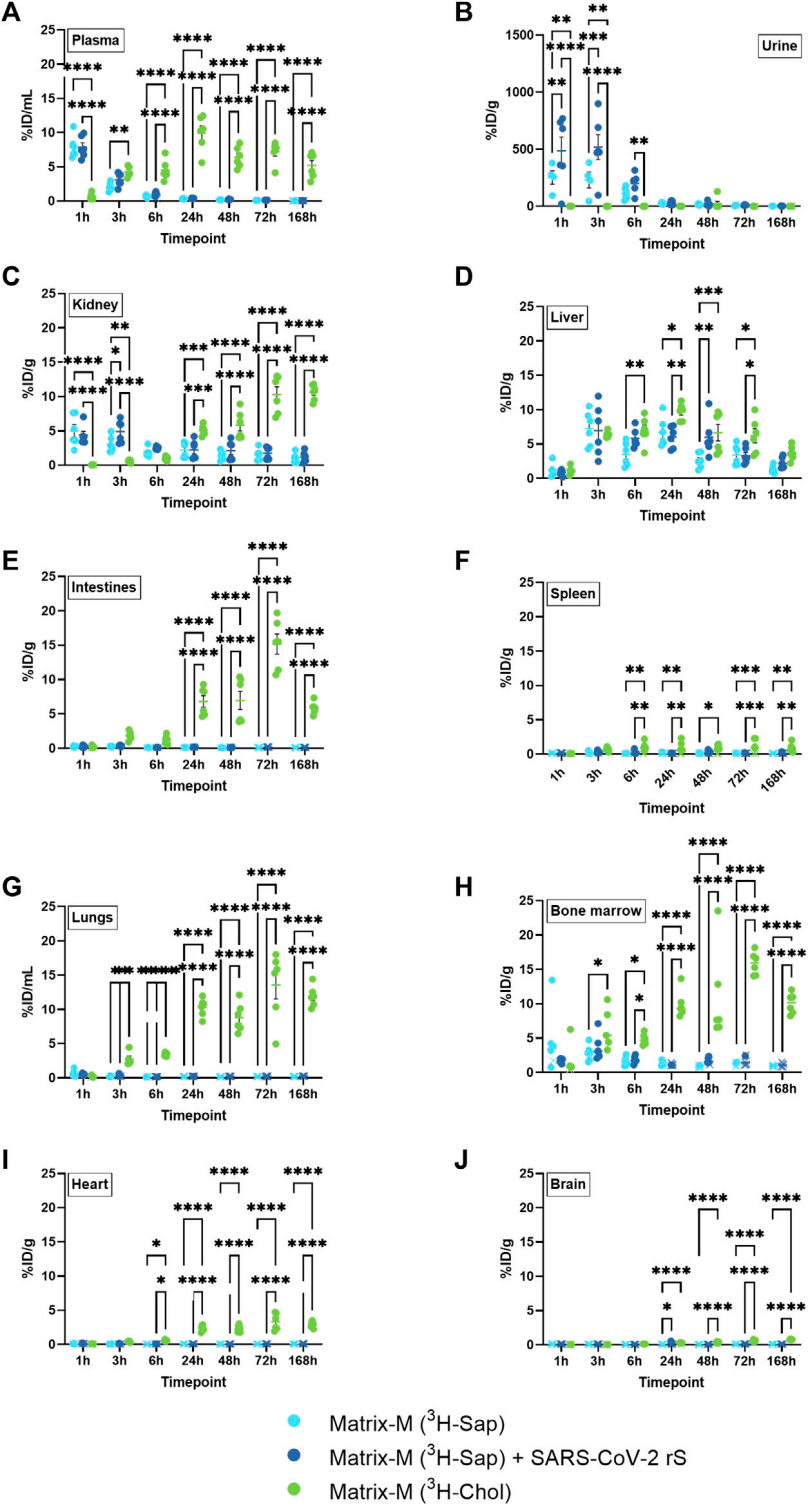
Systemic distribution of saponins and cholesterol. Matrix‐M™ adjuvant was formulated with either radiolabeled ^3^H-saponin or ^3^H-cholesterol, the former administered with or without SARS-CoV-2 rS antigen. The activity was measured by liquid scintillation and expressed as % of injected dose (ID) per mL in plasma and urine **(A,B)** or gram tissue (kidney, liver, intestines, spleen, lungs, bone marrow, heart, and brain) **(C–J)**. Activity (DPM) below the LOQ (703 DPM) was set to 703 DPM and the %ID/g tissue was determined accordingly. Each datapoint represents an individual animal, the horizontal bar denotes the mean+/-standard deviation. Datapoints below the LOQ are shown as X. The data were analyzed separately for each tissue and timepoint by two-way ANOVA with Tukey’s multiple comparisons test. Statistically significant differences between groups are denoted with *, *p* < 0.05; **, *p* < 0.01; ***, *p* < 0.001; ****, *p* < 0.0001. *n* = 4–6.

Body fluids and non-local tissues were studied to understand both the systemic distribution of Matrix-M™ adjuvant and its constituent compounds, and to indicate possible excretion pathways and their time course. The retrieved relative radioactivity of labeled saponins differed from that of cholesterol at most timepoints in plasma, urine, and kidney (Figures 3A–C). The relative activity of labeled saponins in plasma, urine, and kidney peaked at 1–3 h p.i. followed by a rapid decline at 6 h p.i. (Figures 3A–C; Supplementary Figure S2A, C). In contrast, lower relative radioactivity of cholesterol was detected early in plasma and kidney followed by an increase to a plateau at around 5%–20% ID/g at 24 h p.i. which persisted until the last measurement at 168 h p.i., comparable to findings in the non-draining LN (Figures 3A–C; Supplementary Figure S2B, D, 3). Barely detectable activity of cholesterol was observed in urine, which is as expected for a lipophilic compound such as cholesterol. Of note, the presence of SARS-CoV-2 antigen in the immunization led to an increased relative activity of saponins in urine at 1 h and 3 h p.i. and in kidney at 3 h p.i. (Figures 3B, C). In contrast, the absence or presence of antigen had no effect on the saponin activity in plasma, liver, intestines, spleen, lungs, bone marrow, heart, and brain at any study timepoint (Figures 3A, D–J).

A unique pattern of relative activity of saponins in relation to cholesterol was found in the liver with low levels at 1 h p.i. for both labeled compounds, which then increased to a similar activity for both at 3 h p.i. followed by a slightly higher relative activity of cholesterol between 6 and 72 h p.i. (Figure 3D).

The bone marrow exhibited a peak of saponins at 3 h and 6 h p.i., which declined at 24 h p.i. (Figure 3H; Supplementary Figure S2C). Low relative activity of saponins, partially below LOQ, were found in intestines, spleen, lungs, heart, and brain (Figures 3E–G, I, J). Intestines, lungs, and bone marrow showed an increase in the relative activity of cholesterol, reaching a plateau of around 5–20 %ID/g tissue at 24 h p.i., which was stable through 168 h p.i., with comparable levels found in non-draining LN, liver, and kidney (Figures 3E, G, H; Supplementary Figure S2D). A similar pattern with plateauing cholesterol activity from 24 h p.i. was found in spleen, heart, and brain, albeit at lower levels (Figures 3F, I, J; Supplementary Figure S2D).

Thus, these analyses further suggest a rapid and substantial disassembly of Matrix particles *in vivo* as demonstrated by a different biodistribution of saponins and cholesterol. Saponins in Matrix-M™ adjuvant do not accumulate systemically but are rapidly excreted via the kidneys into urine. In contrast, systemic cholesterol levels reached a plateau at around 24 h p.i. that remained stable until the end of measurement (168 h p.i.). This suggests that, as the particle disassembles, labeled cholesterol enters the body’s cholesterol recycling pool before gradual excretion/metabolism.

### 3.4 Biodistribution to the reproductive tract

**FIGURE 4:**
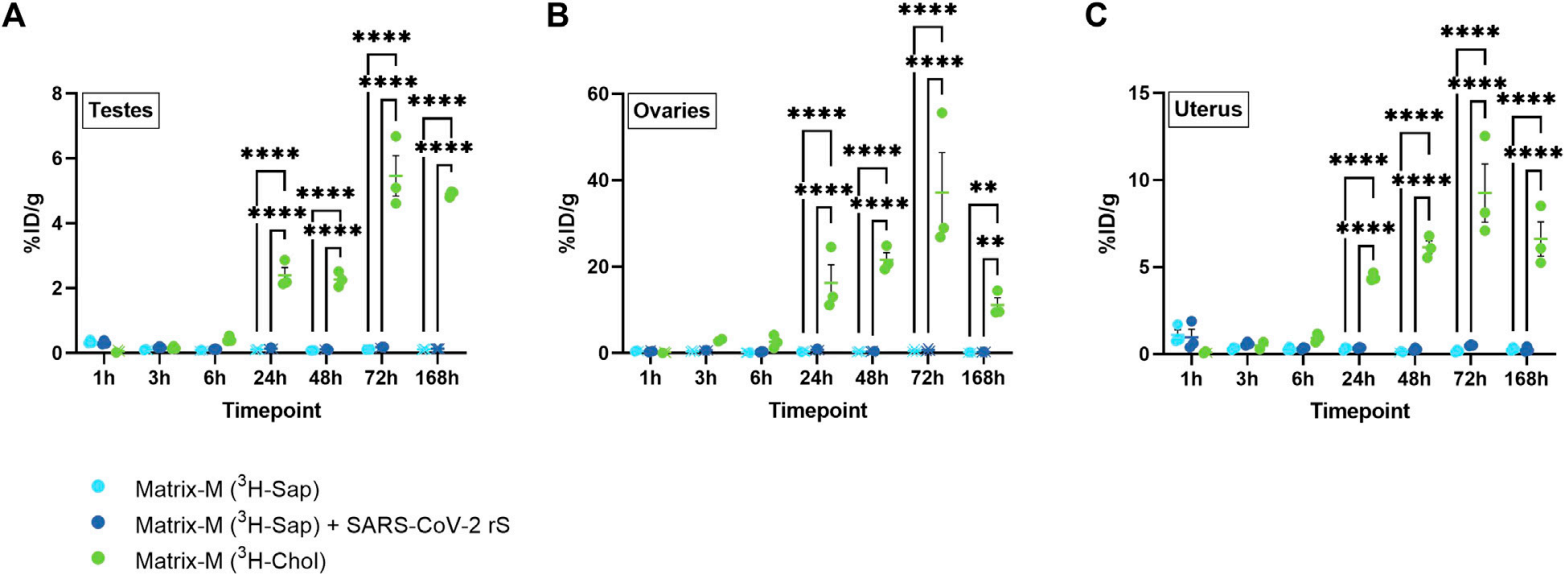
Distribution of saponins and cholesterol to reproductive organs. Matrix‐M™ adjuvant was formulated with either radiolabeled ^3^H-saponin or ^3^H-cholesterol, the former administered with or without SARS-CoV-2 rS antigen. The activity was measured in testes (males) **(A)** or ovaries **(B)** and uterus (females) **(C)** by liquid scintillation and expressed as % of injected dose (ID) per gram tissue. Activity (DPM) below the LOQ (703 DPM) was set to 703 DPM and the %ID/g tissue was determined accordingly. Each datapoint represents an individual animal, the horizontal bar denotes the mean+/-standard deviation. Datapoints below the LOQ are shown as X. The data were analyzed separately for each tissue and timepoint by two-way ANOVA with Tukey’s multiple comparisons test. Statistically significant differences between groups are denoted with *, *p* < 0.05; **, *p* < 0.01; ***, *p* < 0.001; ****, *p* < 0.0001. *n* = 2–3.

To evaluate the biodistribution of labeled saponins and labeled cholesterol in organs of the reproductive tract after Matrix-M™ adjuvant injection, testes from male mice as well as ovaries and uterus from female mice were analyzed. The relative activity of saponins was low in all three organs with the highest relative activity detected at 1 h p.i., which then declined at 3 h p.i. to remain low until the last measurement at 168 h p.i. (Figure 4; Supplementary Figure S4). Adding the SARS-CoV-2 rS antigen to the adjuvant had no effect on the relative saponin activity in the reproductive organs of either males or females. The pattern of the relative cholesterol activity in all three organs reached a plateau starting from 24 h p.i. with the highest relative activity of cholesterol detected in the ovaries. Overall, the saponin and cholesterol distribution patterns to the reproductive tract were comparable to those found in the other analyzed solid organs.

## 4 Discussion

Monitoring the biodistribution of Matrix-M™ adjuvant, a key component of NVX-CoV2373 and several novel vaccine candidates, is an important step in elucidating Matrix-M™ adjuvant MoA and safety profile. Here, using Matrix-M™ adjuvant composed of unlabeled Matrix-C™ particles and Matrix-A™ particles containing either radiolabeled saponins or radiolabeled cholesterol, we were able to demonstrate a very rapid transfer of Matrix-M™ adjuvant from the injection site and the dLN after intramuscular injection. A sharp reduction of both labeled components was found at the injection site and in the dLN within 48 h, together with a transient increase of saponin counts, but not of cholesterol, in plasma, urine, and kidneys at early timepoints. Low levels of relative cholesterol radioactivity were detected systemically between 24 h and the latest measurement of the study (168 h). In general, the biodistribution of saponins was independent of the presence of the rS nanoparticle antigen in the injection. Of note, this study demonstrates the biodistribution of saponins and cholesterol of Matrix-A™, the main component of Matrix-M™, after immunization with Matrix-M™. Due to the low content of Matrix-C™ in Matrix-M™, radiolabeling of Matrix-C components is not sufficient for detection. Therefore, a slightly different biodistribution profile for Matrix-C™, e.g., in its kinetics cannot be excluded.

### 4.1 Injection site and draining LN distribution of Matrix-M™ adjuvant

The fast removal of saponins and cholesterol from the injection site within 48 h p.i. together with an early distribution of saponins and cholesterol to the dLN is congruent with the assumption that the MoA of Matrix-M™ adjuvant is independent of a depot effect (Bengtsson et al., 2013). Instead, the rapid distribution of saponins and cholesterol to the iliac LN further strengthens the concept of Matrix-M™ adjuvant as “setting the stage” for the antigen in the dLN. These results are in line with a comparison of adjuvant-active and attenuated minimal QS-21 saponin adjuvants demonstrating a preferential localization of the active QS-21 in the draining lymph node at 24 h p.i. (Fernández-Tejada et al., 2014). Of note, in the presented radioactivity data, the iliac LN (and all other LN) from the injected and opposing site were pooled together, most likely diluting the observed effect as only changes at the draining site can be expected.

The present finding of the rapid transfer of Matrix-M™ adjuvant to the dLN is also consistent with previously published data describing Matrix-M™ as a potent inducer of immune cell migration (mainly monocytes, DCs, and neutrophils) to the dLN shortly after injection of Matrix-M™ adjuvant with or without antigen (Reimer et al., 2012; Magnusson et al., 2013). These cells had acquired an activated phenotype as required to initiate antigen specific adaptive immunity; an effect that was more pronounced for Matrix-M™ adjuvant in a head-to-head comparison with Alhydrogel, Freund´s Complete Adjuvant and AS03 at 48 h post subcutaneous immunization (Magnusson et al., 2013).

For other saponin-based adjuvants, a similar immediate distribution to dLN has been demonstrated. Using fluorescently labeled QS-21 saponin incorporated in the liposome-based adjuvant AS01 used together with a fluorescently labeled antigen, Didierlaurent et al. (2014) detected both components in the dLN shortly after injection. In addition, a recent study described an increased lymph flow induced, in a histamine-dependent manner, by saponin-based nanoparticles formulated with phospholipids and monophophoryl lipid A (Silva et al., 2021). However, the exact mechanisms by which Matrix-M™ adjuvant and antigen reach the dLN, e.g., the relative contributions of cellular transport and free flow through the lymph, remain to be investigated in future studies.

### 4.2 Matrix particle integrity and excretion pathways

Although the data presented here show a fast reduction of either of the two labeled Matrix components, saponin and cholesterol, within 48 h in the QF, the more rapid removal of saponins than cholesterol from QF suggests a significant disassembly of the Matrix particles already at the injection site. The strong affinity between saponins and cholesterol is well recognized (Bangham and Horne, 1962; Lövgren and Morein, 1988; Beck et al., 2015; De Groot and Müller-Goymann, 2016) and cholesterol is crucial for the formation of Matrix particles, which are stable on storage in neutral buffers. However, the fate of the Matrix particles *in vivo* is probably different. It is believed that, similar to other saponin-based adjuvants such as ISCOMATRIX and AS01, the Matrix particles target phagocytic cells and are processed by the endosomal/lysosomal pathway, and that such processing is dependent on both the acidification of and the enzymatic activities present in the lysosome (Wilson et al., 2014; Welsby et al., 2017; Stertman et al., 2023). In this intracellular processing, the Matrix particles are likely disassembled at lower pH within the lysosome, liberating the saponins. As has been demonstrated for other saponin-based adjuvants, a key consequence of the liberation of the saponins is the destabilization of the lysosomal membrane (Wilson et al., 2014; Welsby et al., 2017). Lysosomal membrane permeabilization then provides access of the co-administered vaccine antigens to the cytosol, thus allowing cross-presentation by MHC class I molecules and the induction of antigen-specific CD8^+^ T cell responses. Such responses have been demonstrated for Matrix-M™ adjuvanted vaccines in murine models and in humans upon vaccination with NVX-CoV2373 (Bengtsson et al., 2016; Tian et al., 2021; Rydyznski Moderbacher et al., 2022).

In addition, the transient peak detection of saponins but not of cholesterol in plasma, urine, and kidneys before 24 h p.i. confirms the distinct excretion pathways of non-associated cholesterol and saponin and their respective degradation products. In the liver, similar relative saponin and cholesterol activities were detected at 1 and 3 h p.i. However, this does not necessarily imply that Matrix particles are intact in this particular organ, as other studied fluids and tissues indicated at least a partial disassembly of Matrix particles at these timepoints. Saponins and possible degradation products appeared rather quickly in urine with a near complete clearance within 7 days, thus indicating this as a major excretion pathway. In contrast, cholesterol was detected systemically between 24 h and the latest measurement of the study (168 h), although the systemic cholesterol radioactivity was low in comparison to initial activities in the QF and the iliac LN. It can be speculated that at least a part of the labeled cholesterol enters the cholesterol recycling pool as an essential component of cell membranes, which may explain the systemic detection and slower clearance kinetics. Due to technical limitations, it was not possible to analyze feces in this study, which generally represents a major pathway for the elimination of cholesterol.

### 4.3 Effect of antigen on saponin biodistribution and excretion

Generally, no differences in the saponin biodistribution dependent on the presence or absence of the rS nanoparticle antigen were found, as also described for other antigens and minimal QS-21 (Fernández-Tejada et al., 2014). This was not a surprise as, in contrast to the saponin-based adjuvant ISCOM in which the antigen is physically incorporated into the particle, Matrix formulations consist of formulated particles that are mixed with antigen (Lövgren Bengtsson et al., 2011). However, at a few timepoints, the presence of rS antigen in the immunization led to an increased relative activity of saponins in the urine (1 h and 3 h p.i.) and in the kidney (3 h p.i.). As these differences are minor and rare the overall interpretation is that the presence or absence of vaccine antigen does not substantially influence the biodistribution of saponins.

### 4.4 Matrix-M™ adjuvant safety profile

The localized and time-restricted occurrence of Matrix-M™ adjuvant at the injection site and its rapid distribution to the dLN is not only favorable for the efficient induction of a memory response specific for the co-administered antigen but also from a safety profile perspective. The observed rapid clearance of saponins without a general systemic distribution can be incorporated into the benefit-risk assessments of Matrix-M™-adjuvanted vaccines. Of note, no long-term accumulation of saponins in any organ (including the reproductive tract) was observed. It is reasonable to believe that the lack of complete elimination of cholesterol after 168 h can be explained by cholesterol from the Matrix-particles entering the endogenous pool as essential components of cell membranes or metabolites. The described overall favorable safety profile from a biodistribution perspective confirms the results of a mild to moderate and transient reactogenicity of numerous phase 1–3 clinical trials evaluating vaccines containing Matrix-M™ adjuvant against COVID-19, malaria, influenza, or Ebola Virus Disease (Fries et al., 2020; Keech et al., 2020; Datoo et al., 2021; Heath et al., 2021; Shinde et al., 2021; 2022; Dunkle et al., 2022).

## 5 Conclusion

In summary, this biodistribution study on Matrix-M™ adjuvant provides useful insights that match and complete results from previously performed immunologically focused studies and clinical trials. The data demonstrate a rapid transfer of Matrix-M™ to the dLN, followed by a rapid removal of detected saponins and cholesterol from both the injection site and the dLN. Importantly, this transient presence of the adjuvant in the dLN is sufficient for the generation of high titers of neutralizing antibodies and immune memory, and an IgG2a signature consistent with a strong Th1 T cell response. The head-to-head comparison of saponins and cholesterol as critical components of Matrix particles indicate a fast particle disassembly at the injection site, which may be crucial for intracellular events like the lysosomal membrane permeabilization required for the reported CD8^+^ T cell activation in response to Matrix-M™-adjuvanted antigens. To fully understand the MoA of Matrix-M™ adjuvant, future studies should consider a combination of biodistribution and immunological perspectives at the tissue, cellular, and intracellular level.

## Data Availability

The original contributions presented in the study are included in the article/Supplementary Material, further inquiries can be directed to the corresponding author.
