# Supplementary material for: Biodistribution of the saponin-based adjuvant Matrix-M™ following intramuscular injection in mice
Source: Front Drug Deliv. 2023 Nov 6;3:1279710. doi: 10.3389/fddev.2023.1279710 (PMC12363263; doi:10.3389/fddev.2023.1279710)
Supplement: Supplementary file 1 [file DataSheet1.PDF]

Supplementary Figure 1

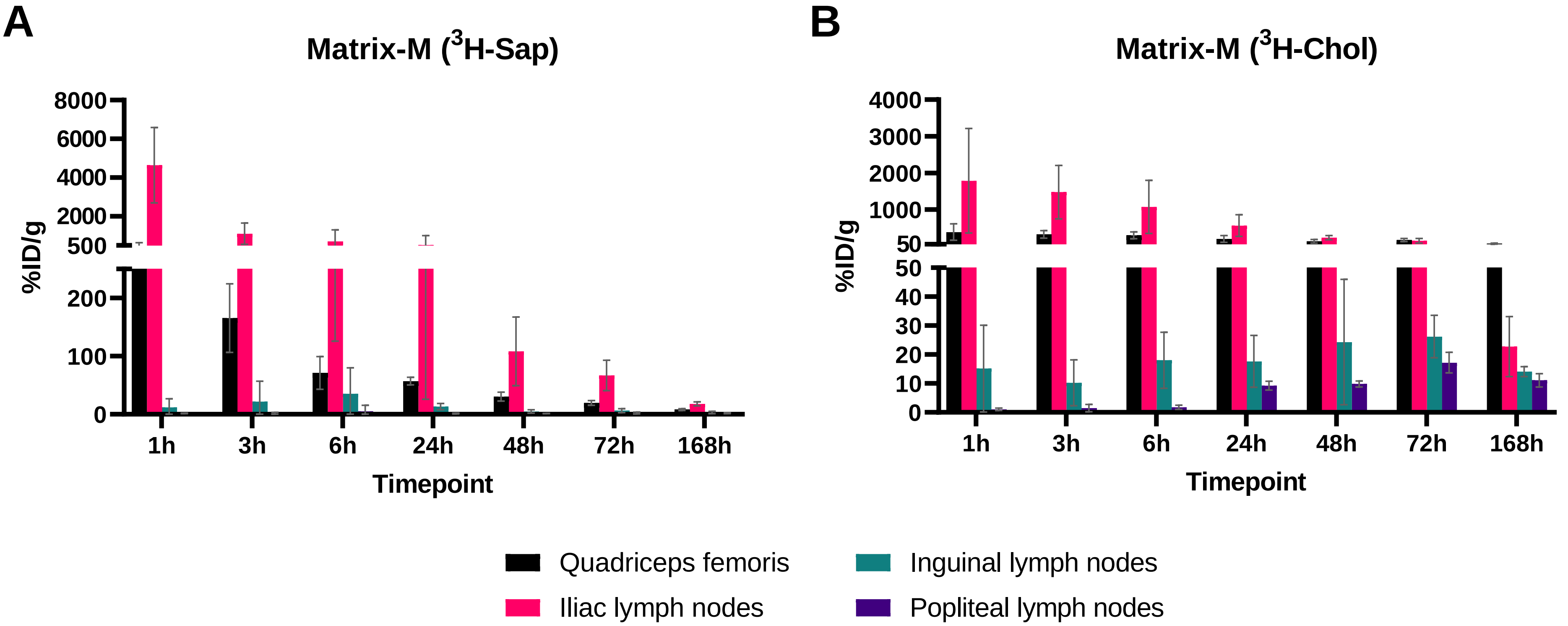

Supplementary Figure S1. (A) The activity of 3H-labeled saponin at the injection site and the draining (iliac, inguinal, and popliteal) lymph nodes demonstrating the different distribution and clearance of saponin over time. **(B)** The activity of 3H-labeled cholesterol at the injection site and the draining (iliac, inguinal, and popliteal) demonstrating the different distribution of cholesterol over time. The data are the same as in Figure 2 here projected as mean +/- SD. n=5-6.

Supplementary Figure 2

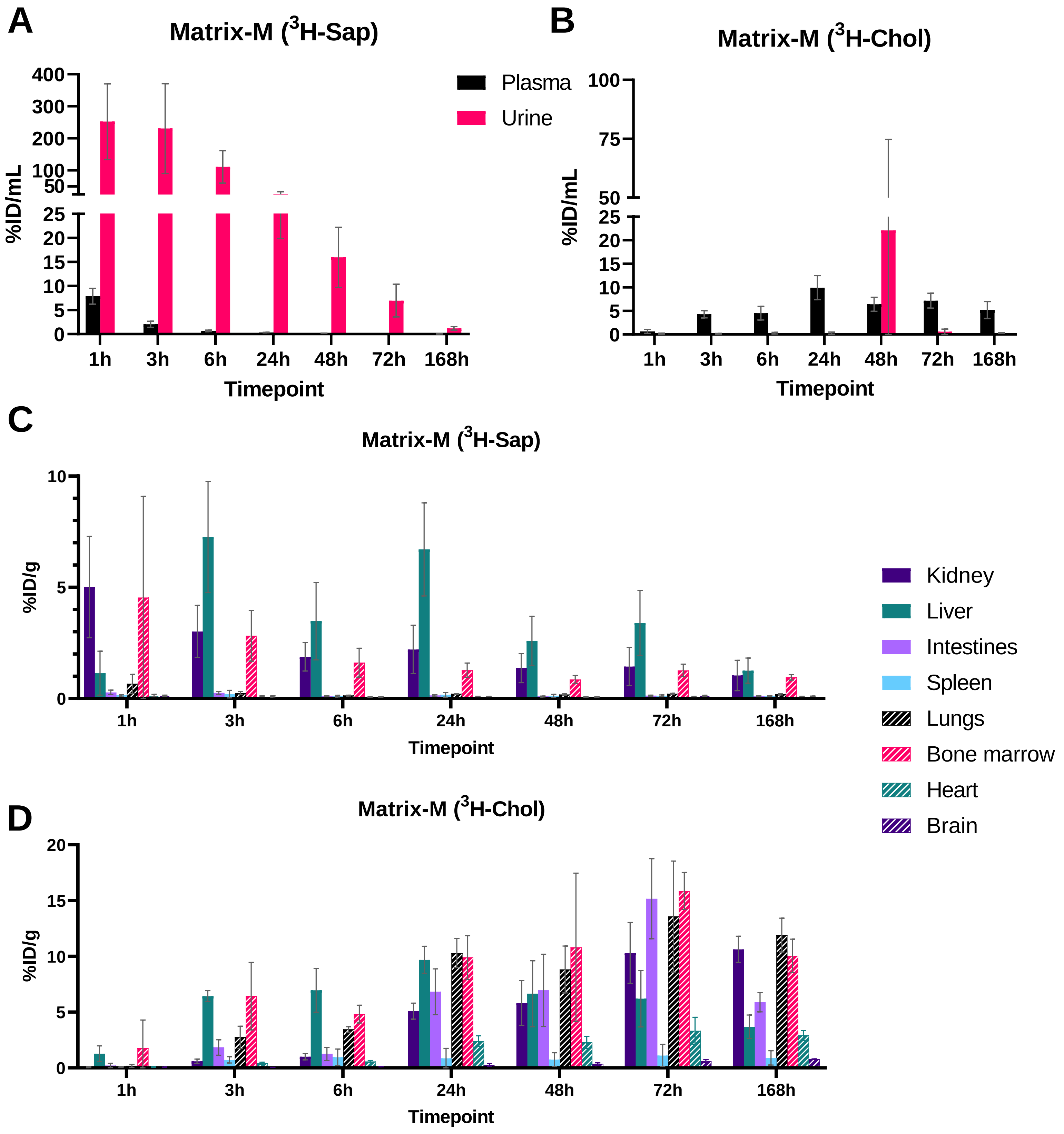

Supplementary Figure S2. (A) The activity of 3H-labeled saponin in plasma and urine, demonstrating the clearance of saponin over time. **(B)** The activity of 3H-labeled cholesterol in plasma and urine, demonstrating how cholesterol distributes to these compartments over time. **(C)** The activity of 3H-labeled saponin in kidney, liver intestines, spleen, lungs, bone marrow, heart, and brain, demonstrating the systemic distribution and clearance of saponin over time. **(D)** The activity of 3H-labeled cholesterol in liver, kidney, intestines, spleen, lungs, bone marrow, heart, and brain, demonstrating how cholesterol distributes to these compartments over time. The data are the same as in Figure 3, here projected as mean +/- SD. n=4-6

Supplementary Figure 3

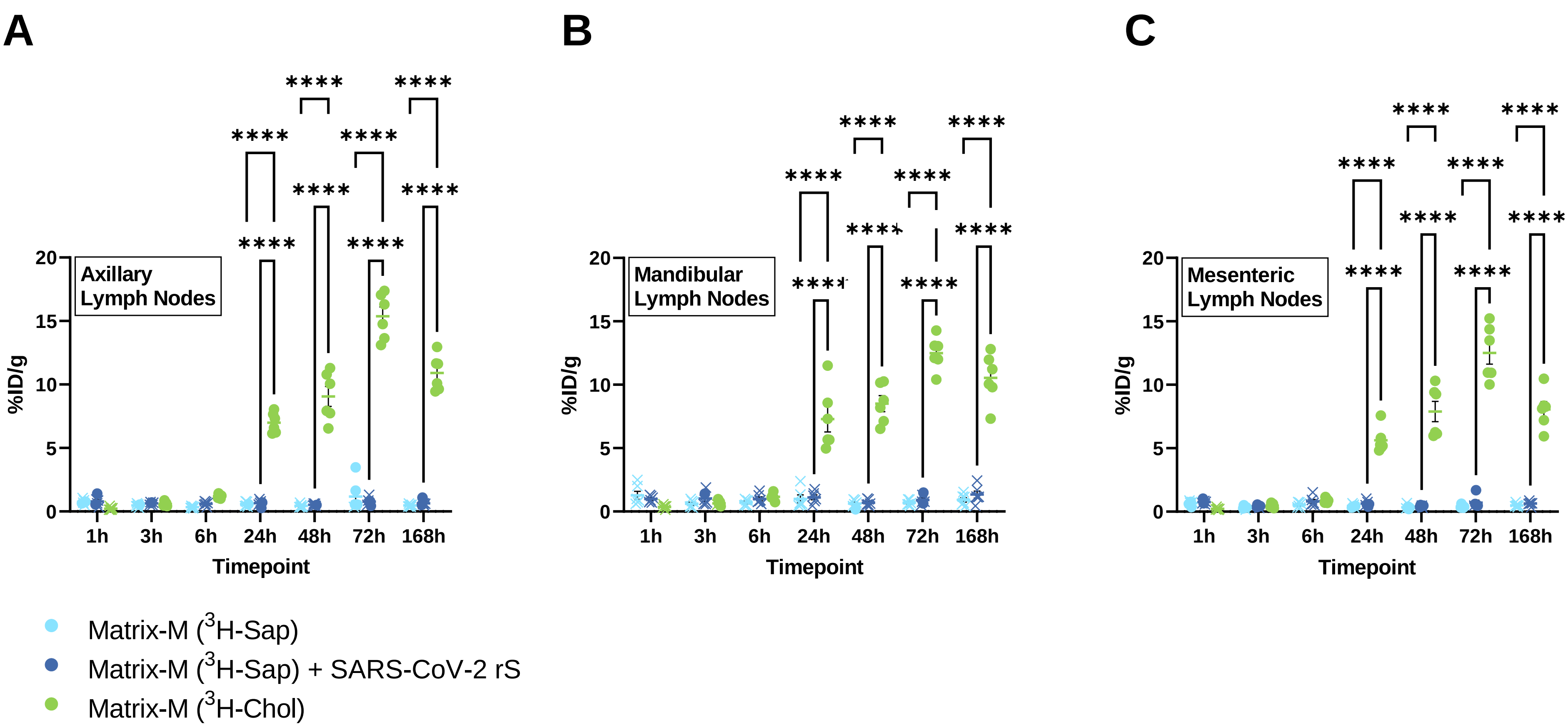

Supplementary Figure S3. Matrix-M adjuvant was formulated with either radiolabeled (3H-) saponin or 3H-cholesterol, the former administered with or without SARS-CoV-2 rS antigen. The activity was measured in the axillary **(A)**, mandibular **(B)**, and mesenteric **(C)** lymph nodes (non-draining; pooled left and right) by liquid scintillation and expressed as % of injected dose (ID) per gram tissue. Activity (DPM) below the LOQ (703 DPM) was set to 703 DPM and the %ID/g tissue was determined accordingly. Datapoints below the LOQ are shown as X. The data were analyzed separately for each tissue and timepoint by two-way ANOVA with Tukey's multiple comparisons test. Statistically significant between groups are denoted with *, p<0.05; **, p<0.01; ***, p<0.001; ****, p<0.0001. n=5-6.

Supplementary Figure S4

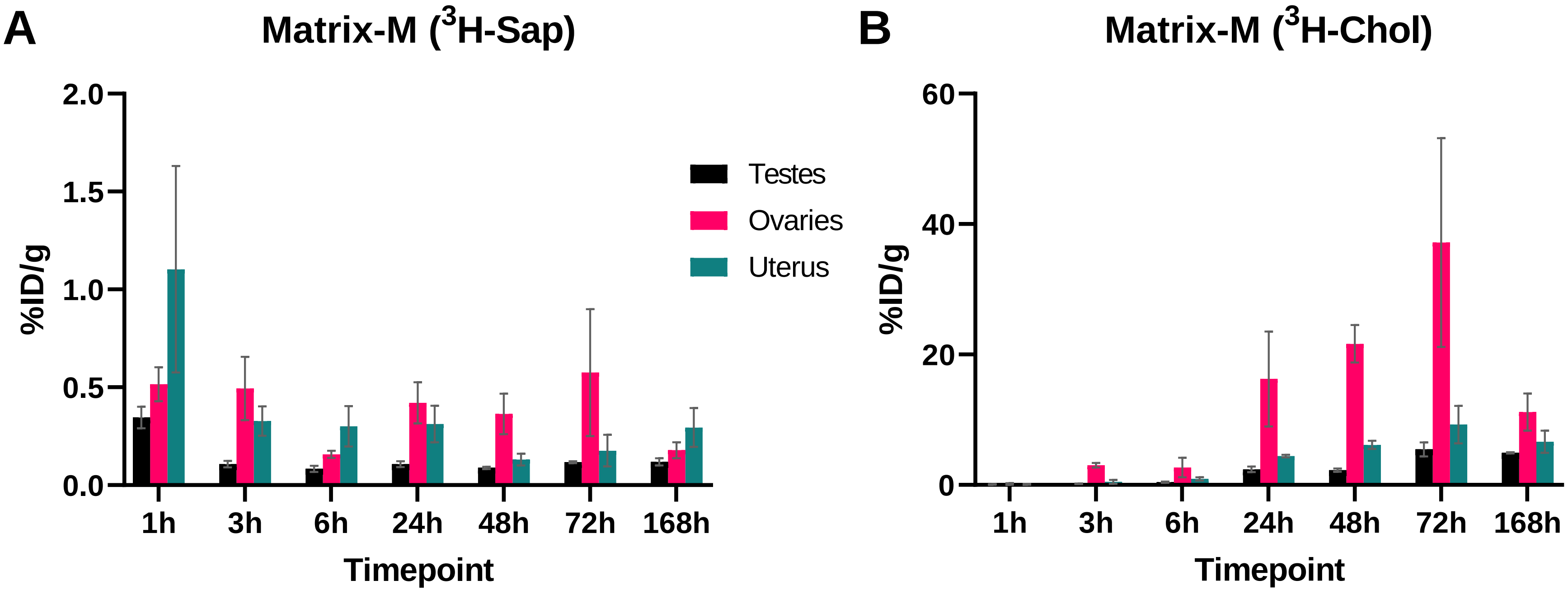

Supplementary Figure S4. (A) The activity of 3H-labeled saponin in testes, ovaries, and uterus, demonstrating the distribution of saponin to the reproductive organs over time. **(B)** The activity of 3H-labeled cholesterol in testes, ovaries, and uterus, demonstrating how cholesterol distributes to the reproductive organs over time. The data are the same as in Figure 4, here projected as mean +/- SD. n=2-3.
